# Biases and limitations of Global Forest Change and author-generated land cover maps in detecting deforestation in the Amazon

**DOI:** 10.1371/journal.pone.0268970

**Published:** 2022-07-06

**Authors:** Eva Kinnebrew, Jose I. Ochoa-Brito, Matthew French, Megan Mills-Novoa, Elizabeth Shoffner, Katherine Siegel

**Affiliations:** 1 Department of Ecosystem Science and Sustainability, Colorado State University, Fort Collins, Colorado, United States of America; 2 Geography Graduate Group, University of California, Davis, California, United States of America; 3 Fundación EcoCiencia, Quito, Ecuador; 4 Department of Environmental Sciences, Policy, and Management, University of California, Berkeley, California, United States of America; 5 Energy and Resources Group, University of California, Berkeley, California, United States of America; 6 Department of Geography, University of Washington, Seattle, Washington, United States of America; 7 Department of Ecology & Evolutionary Biology, University of Colorado, Boulder, Colorado, United States of America; Indiana State University, UNITED STATES

## Abstract

Studying land use change in protected areas (PAs) located in tropical forests is a major conservation priority due to high conservation value (e.g., species richness and carbon storage) here, coupled with generally high deforestation rates. Land use change researchers use a variety of land cover products to track deforestation trends, including maps they produce themselves and readily available products, such as the Global Forest Change (GFC) dataset. However, all land cover maps should be critically assessed for limitations and biases to accurately communicate and interpret results. In this study, we assess deforestation in PA complexes located in agricultural frontiers in the Amazon Basin. We studied three specific sites: Amboró and Carrasco National Parks in Bolivia, Jamanxim National Forest in Brazil, and Tambopata National Reserve and Bahuaja-Sonene National Park in Peru. Within and in 20km buffer areas around each complex, we generated land cover maps using composites of Landsat imagery and supervised classification, and compared deforestation trends to data from the GFC dataset. We then performed a dissimilarity analysis to explore the discrepancies between the two remote sensing products. Both the GFC and our supervised classification showed that deforestation rates were higher in the 20km buffer than inside the PAs and that Jamanxim National Forest had the highest deforestation rate of the PAs we studied. However, GFC maps showed consistently higher rates of deforestation than our maps. Through a dissimilarity analysis, we found that many of the inconsistencies between these datasets arise from different treatment of mixed pixels or different parameters in map creation (for example, GFC does not detect reforestation after 2012). We found that our maps underestimated deforestation while GFC overestimated deforestation, and that true deforestation rates likely fall between our two estimates. We encourage users to consider limitations and biases when using or interpreting our maps, which we make publicly available, and GFC’s maps.

## Introduction

Deforestation in and around protected areas (PAs) is a persistent threat to ecosystems and human livelihoods [1, 2] https://www.zotero.org/google-docs/?ntlTvF. PAs within tropical forests experience particularly high deforestation rates due in part to the suitability of land for agricultural production [3–5] https://www.zotero.org/google-docs/?ZcL6SV. Tracking land use change in these areas using remote sensing is important to understand deforestation trends and dynamics [6, 7]. For instance, spatial land use change data can indicate what areas are of potential deforestation risk (e.g., due to logging or agricultural frontiers), which is key information used by government officials, conservation organizations, and researchers in designing management and policies. Due to the highly local dynamics of PA deforestation and high conservation interest in these areas, it’s important to draw from land cover products that are accurate and effectively represent land use change trends.

Researchers use a variety of methods and spatial datasets to detect land use change in and around PAs. Many studies generate remote sensing products themselves [8, 9], while others use readily available land cover maps [10–12]. Generating one’s own land cover maps has benefits including having more say over the land cover map’s attributes, such as land cover classes and the map’s time range. Available land cover map products, such as the Global Forest Change dataset [13], MODIS Land Cover [14], or SoilGrids [15], are extremely useful because they allow for large scale analysis and make these analyses accessible to scholars who might not have the skills, resources, or time to create accurate remote sensing products of their own.

The Global Forest Change (GFC) data [13] is particularly well known and highly utilized for assessments of land use change in and around PAs [1, 10–12, 16], thereby contributing to understanding of PA deforestation on a global level. GFC has mapped forest loss since 2000 and is valuable due to its high resolution (30m), standardized classes, yearly updates, and convenient and cost-free use (such as availability through Google Earth Engine). This wealth of data and ease of use has contributed to GFC being used to inform policy and management. For instance, GFC is an input in Global Forest Watch, a web tool that many organizations use to track land use change [17], and has been considered for use informing REDD+ policy decisions [18]. GFC is also used to calculate carbon budgets [19, 20], thereby informing climate change policy.

However, a number of studies have found accuracy issues for this dataset, such as lower accuracy for some ecosystems [21, 22], underestimation of forest cover [20, 22], and moderate inaccuracies in identifying the year of deforestation [23]. While some of these errors in fact reflect stated limitations of this product [24], assessing both the biases and limitations of GFC are valuable to most accurately convey land use trends.

To test the accuracy of GFC and other land cover maps, many studies have quantified differences between maps or compared maps to ground-truthed data [22, 25–28]. However, few papers comprehensively address *why* biases occur, often termed uncertainty or dissimilarity analyses [29]. There are many reasons for dissimilarity between datasets, including classification errors, different treatment of mixed pixels, and differences within algorithm parameters, all of which can affect classification imagery results and present certain biases. Dissimilarity analyses can help expose each map’s biases, which ultimately informs researchers and managers on the strengths and limitations of different products, allows for correction, and contextualizes past and future studies that use these datasets. Furthermore, using multiple products can help ascertain land use transitions by corroborating trends or unveiling nuance through their dissimilarities.

In this study, we created Landsat-derived land cover maps and compared them with GFC products. We use these two products to 1. Understand how these two land cover maps represent land use change trends in and around PAs; 2. Quantify differences between the two land cover maps; and 3. Conduct a dissimilarity analysis to detect why differences occur and to quantify bias.

We centered our analysis on land use changes between 2008 and 2018 in three PA complexes in the Amazon Basin: Amboró and Carrasco National Parks in Bolivia, Jamanxim National Forest in Brazil, and Tambopata National Reserve and Bahuaja-Sonene National Park in Peru. We chose three PA complexes in the Amazon Basin because this area is characterized by high rates of land use change, including deforestation related to agriculture, logging, and mining. The escalating rate of deforestation in the Amazon basin is troubling due to high species endemism, biodiversity, sites of cultural importance, Indigenous communities, its role in global climate systems, and high carbon storage found here [30–33]. Accurate and detailed understanding of land use change in the Amazon–and the creation of publicly-available remote sensing data whose biases are explicit–can aid in communication around land cover change in the Amazon, feed into future research, such as modeling efforts, and inform policy and management decisions.

## Methods

### Case study selection

We chose our three case study sites, Amboró and Carrasco National Parks in Bolivia, Jamanxim National Forest in Brazil, and Tambopata National Reserve and Bahuaja-Sonene National Park in Peru, because they are similar sizes, exhibit high levels of deforestation compared to other PAs in the Amazon Basin (Table 1), and are associated with different sets of deforestation drivers. The Bolivian and Peruvian sites each include two adjacent PAs, and the study site in Bolivia also contains the integral management area around Amboró National Park. Including all three case studies affords us the opportunity to examine the performance of different land cover products in capturing land use change patterns in a variety of contexts.

**Table 1:** General characteristics of our three case study sites. We calculated deforested area here using the Global Forest Change dataset.

	Amboró & Carrasco National Parks	Jamanxim National Forest	Tambopata National Reserve & Bahuaja-Sonene National Park
**Country**	Bolivia	Brazil	Peru
**Total area (km** ^**2**^ **)**	13,598	13,242	14,169
**Total deforested area 2000–2018 (km** ^**2**^ **)**	526	1,573	93

Amboró and Carrasco National Parks face deforestation pressures associated with the migration of Andean settlers and expansion of small-scale agriculture [34–36]. The multi-use Integrated Management Natural Area (IMNA), which surrounds Amboró National Park, was created in 1995 in response to social unrest following the controversial expansion of park boundaries in 1991 [34]. The IMNA allows for multiple land uses, but the rest of the park is strictly protected by law. Both Amboró and Carrasco are overseen by the Bolivian National Service for PAs with international financial assistance.

Jamanxim National Forest in Brazil was part of a matrix of PAs created as part of a sustainable development planning initiative to limit the deforestation associated with highway BR-163. Jamanxim, however, has faced pressures for downsizing and downgrading related to both pending infrastructure projects and land claims made within the PA [37, 38], which is concerning because this area has already suffered significant deforestation and forest degradation from logging and ranching [39].

Tambopata National Reserve and Bahuaja-Sonene National Park draw on a participatory model of conservation governance shaped by the incorporation of indigenous communities and small farmers in the conservation planning process for an area with a history of ecotourism [40]. Nevertheless, the Madre de Dios region of Peru and Tambopata in particular have also experienced a surge in small-scale gold mining during the study period of this research, which threatens the conservation area and its buffer zone [8, 16, 41, 42].

For all three case study sites, we included the area within the PA boundaries (acquired from the World Database on Protected Areas [43]) and a 20 km buffer around the PA. This allowed us to capture dynamics in the area surrounding the PAs, where phenomena such as leakage can impact land use processes [44].

### Remote sensing within case study sites

#### Image compositing and cloud masking

We used Google Earth Engine [45] to create cloud-free image composites of each case study. These composites encompassed pixels from Landsat 5 (TM) [46], Landsat 7 (ETM+) [47], and Landsat 8 (OLI) [48] 30m Surface Reflectance (SR) datasets. Unlike top of atmosphere (TOA) data, SR products have passed through an atmospheric correction process and thus provide reflectance values as they would be measured at ground level.

In our compositing algorithm, we restricted data collection to the dry season (15 May to 15 October) for 2008 and 2018. We masked clouds by pixel quality analysis using the CFMask algorithm within Google Earth Engine [49]. The compositing algorithm prioritized Landsat 8 imagery for the 2018 imagery and Landsat 5 for the 2008 imagery.

We collected ancillary environmental data to enhance land classification accuracy. This data included elevation from the SRTM Digital Elevation Data at 30m, Enhanced Vegetation Index (EVI) (calculated with bands from our cloud-free composites), and difference in seasonal EVI [50] between wet and dry seasons. These additional data were added as bands to the remote sensing data. Using the difference in seasonal EVI allows for better differentiation of land covers that have larger seasonal phenological differences, like agriculture, from land covers that have smaller seasonal phenological differences, like forests [51, 52]. To calculate EVI difference for a specific year, we created cloud-free imagery and calculated EVI for the dry season (mid-May to mid-October) and the wet season (mid-October to mid-May, advancing into the subsequent year). We then subtracted the dry season EVI images from the wet season and took the difference to find absolute change.

#### Training data collection

We selected 1000 randomly positioned points within each case study (total 3000) for training data collection. Around each point, we created a 250x250m window. Using the online platform Geosurvey (Quantitative Engineering Design; https://qed.ai), we classified land into categories: agriculture, forest, bare soil, urban, wetland, desert, and water. Geosurvey uses satellite image sources including Bing Aerial, Google Hybrid, and Mapbox to present high resolution imagery (1m or finer) and allow users to manually classify imagery by drawing polygons around each land cover present within a training window. We considered pasture and cropland as agriculture. In some instances, it was unclear whether land was pasture or very early successional forest. In these cases, we classified it as agriculture. Areas that had been recently logged but had little detectable vegetation were classified as bare soil. We classified river banks as bare soil and all buildings as urban.

While Geosurvey generally presents the most up-to-date images, an important limitation is that it does not list the acquisition date of the imagery shown or allow users to look at historical imagery. Because we classified 2018 imagery in 2019, the Geosurvey imagery should generally correspond to our composited images. However, in the case that the land cover patterns changed between the time of the Geosurvey images and our 2018 imagery, there is the potential for classification errors. We attempted to reconcile this by comparing potentially problematic polygon windows with our 2018 (much coarser resolution) cloud-free composites.

#### Supervised classification

Using the spectral imagery with added elevation, EVI, and EVI seasonal difference bands and training polygons from Geosurvey, we ran a supervised classification in R version 3.6.2 (R Core Team 2021) using a random forest algorithm with 100 trees [53–55]. After looking at the initial results of the classification, we found sub-optimal classification results for agriculture, bare soil, urban, wetland, and water land cover classes. This is due to a disproportionately high number of forest points (forest was the most common land use in all case study sites). Therefore, to partially reconcile the bias towards forest training data, in Google Earth Engine we collected approximately 10–100 more polygons (250x250m) in the land cover classes agriculture, bare soil, urban, water, and, only in the Peru case study, wetland. This ensured that we had at least 50 polygons (though sometimes up to several hundred polygons) for each major land cover in each case study. These extra classified polygons were randomly distributed across the case studies to avoid possible issues of spatial correlation of nearby polygons, and the inclusion of these extra polygons improved classification results. Our resulting final land cover maps are used in the subsequent analyses and are also made publicly available for future use (see S1 Table).

#### Accuracy of remote sensing products

To assess the accuracy of our maps we applied a 10 k-fold cross validation to obtain a sample confusion matrix comparing reference (training data) and predicted land cover classifications for each of our study areas. We then transformed these sample matrices to estimated population matrices using equation 1 from Pontius et al. (2011) [56]. Calculating estimated population matrices achieves a less biased understanding of accuracy, reflecting conditions over the entire study area rather than simply in the sampled areas. From the estimated population matrices, we calculated overall quantity and allocation difference using the overallQtyD and overallAlloD functions from the diffeR package in R [57]. Quantity difference is the difference in the total amount of pixels (regardless of where they are located) within land cover classes between reference and predicted data. Allocation difference takes into account the spatial disagreement (error in the position) of pixels. We additionally derived user’s and producer’s accuracy measures from our estimated population matrices [58], which reflect commission and omission errors, respectively. User’s accuracy is the number of correctly classified samples of class *A* divided by the total number of samples classified as *A*. Therefore, user’s accuracy is also referred to as “reliability” because the map user is interested in how well the map represents the on-the-ground reality. Producer’s accuracy is the number of correctly classified samples of class *A* divided by the total number of *A* reference samples. Here, the map producer is interested in how accurate a specific land cover is classified. We provide overall quantity and allocation difference and producer’s and user’s accuracies for the 2018 land cover maps only (these are the maps for which we have training data).

### Global Forest Change data collection

We downloaded GFC data [13] within case study areas in Google Earth Engine. To only capture deforestation occurring between 2008 and 2018, we selected the “LossYear” band, which indicates what years pixels were deforested, and used an expression to only include pixels with a loss year between 2008 and 2018 (with all other pixels assigned NA, indicating no change between 2008–2018). The GFC dataset also includes a band for “forest gain.” However, this data is not categorized by year and encompasses gain between the years 2000–2012, and thus does not address reforestation that occurred in the last 6 years of our study period. We used the forest gain band to check for potential reforestation between 2008–2012. If a pixel was deforested between 2008 and 2012, but was marked as reforested, we counted it as “no change.” We reclassified all data into a binary classification including “deforested” and “no change” pixels, representing change between 2008–2018.

### Analysis of trends

To analyze land change trends between 2008–2018 in our maps, we calculated percent pixel change to and from each land cover class. We visualized transitions with Sankey graphs using the ggalluvial package [59]. Sankey graphs show the proportion of data (pixels) transitioning between each set of land covers. For the GFC dataset, which has binary “deforested” and “no change” classes, we reported the percent of pixels that were deforested between 2008 and 2018. We did all analyses separately for pixels within the PA boundaries and pixels in the 20 km buffer around the PAs.

### Comparison of Our and Global Forest Change’s Land Cover Maps

#### Overlap analysis

To directly compare our classified maps with the GFC maps, we simplified our remote sensing maps into binary “deforested” versus “no change” classes. We considered “deforested” transitions as a change from a natural land cover (forest, wetland, or desert) to an anthropogenic land cover (agriculture, urban, bare soil, and water). We considered water an “anthropogenic” land cover because forest to water transitions sometimes represent the creation of mines, which is a common land use transition in parts of the study area [8]. All other transitions (such as agriculture to bare soil or bare soil to urban) were considered “no change.” We additionally considered transitions between desert and bare soil as “no change” due to the difficulty of our algorithms in distinguishing between the two, which caused a falsely high percent of “deforested” transitions. This binary classification of our seven land classes and their transitions is not without fault, but we believe it is a reasonable way to categorize the transitions. “Reforested” areas were also treated as “no change” in order to fairly and directly compare to GFC.

To first calculate dissimilarity between our maps and GFC’s maps for each case study, we created a confusion matrix between the two maps and again computed quantity and allocation differences to obtain us a baseline understanding of how these maps differ. This confusion matrix also allowed us to determine detailed information on the nature of agreements and disagreements between the two maps. Specifically, we determined 1) where the maps agreed there was no deforestation, 2) where the maps agreed there was deforestation, 3) where only GFC detected deforestation, and 4) where only our maps detected deforestation. To spatially portray these changes, we assigned the rasters for our and GFC’s maps slightly different values and subtracted them. In the Bolivian study site, while there were points where desert was deforested (desert to agriculture transitions), we do not include this in our analyses or visual comparison with GFC because GFC was not designed to capture deforestation of desert ecosystems (desert areas are mostly registered as “No Data” within the GFC dataset, perhaps because they generally do not have vegetation taller than 5m, which is the GFC criteria for “forested” areas) [13].

#### Dissimilarity analysis

To further understand *why* there were differences between our classified maps and the GFC maps, we carried out a detailed analysis on areas of disagreement. We did not include desert pixels in this analysis, due to the aforementioned limitation of GFC in detecting deforestation of desert ecosystems. For each case study, we randomly chose 200 pixels where our maps and the GFC disagreed on whether there was net deforestation between 2008–2018. For each of the 600 total pixels, we used high resolution monthly composite imagery from PlanetLabs [60] to zoom into a 30x30m window, representing the Landsat pixel size. We first identified the land cover for May 2018 (corresponding to the beginning of the time range that we used to composite 2018 satellite imagery), classifying imagery into the 7 classes used in our classification scheme. If the 30x30m window was not composed of more than 80% of one land cover, we classified it as “mixed.”

We then categorized pixels by inducing possible explanations for dissimilarity. To fully understand the context around points, we looked forward and backwards two years (2016 through 2020) to understand dynamics or timing of change (such as history of deforestation, or to ascertain whether an area was actively reforesting). We additionally used our remotely sensed image classifications for 2008 and 2018 as well as GFC data including forest gain, the year of forest loss, and the percent forest cover in 2000 to contextualize differences. For forest cover in 2000, we considered pixels with tree covers greater than 70% as “forested” [61]. The explanations for dissimilarity were induced by EK, who looked through 150 points (around 50 per case study) and determined 6 main reasons. The remaining 450 points were classified by both EK and MF into the 6 categories.

We analyzed dissimilarity by tallying, for each protected area complex, how many points fell into each explanation for dissimilarity and, where relevant, which dataset better represented land cover on the ground. For most explanations for dissimilarity, neither map was necessarily right or wrong, and instead the disagreement reflected different algorithm parameters or differing treatment of difficult land covers (like mixed pixels or early successional forest).

## Results

### Remote sensing accuracy

Our classification returned over 90% overall accuracy for all the three sites (with quantity and allocation differences highest in Bolivia and lowest in Peru; Table 2). User’s and producer’s accuracy values showed that forest was accurately classified for all case studies (greater than 94% user’s and producer’s accuracy). Agriculture, bare soil, desert (for Bolivia and Peru), and wetland were also fairly well-classified, with above 70% accuracy for user’s and producer’s values, and generally higher values for user’s accuracy (Table 2). Our classification had low accuracy for urban cover in Bolivia and Brazil (between 50–70% producer’s accuracy), and extremely low accuracy for water in Bolivia (with only 19.9% producer’s accuracy). Urban cover was not common in the case studies, making up less than 0.1% of the total area. Additionally, water cover made up less than 0.01% of the area in the Bolivia site.

**Table 2:** Validation results for our classification maps.

	Bolivia		Brazil		Peru	
Overall quantity difference	2.88%		1.74%		0.93%	
Overall allocation difference	3.99%		1.51%		0.50%	
Overall accuracy (100 minus the combined quantity and allocation difference)	93.13%		96.75%		98.57%	
Accuracy by land cover class	*User’s*	*Producer’s*	*User’s*	*Producer’s*	*User’s*	*Producer’s*
1. Agriculture	91.7%	80.9%	93.9%	81.0%	95.0%	73.2%
2. Forest	94.2%	98.1%	97.4%	99.4%	98.8%	99.8%
3. Bare soil	86.6%	85.5%	77.4%	76.1%	77.3%	70.2%
4. Urban	81.0%	67.2%	83.7%	56.2%	91.9%	71.9%
5. Water	59.5%	19.9%	96.1%	96.2%	98.5%	88.1%
6. Desert	92.0%	84.9%	*NA*	*NA*	94.7%	97.1%
7. Wetland	90.0%	74.1%	95.8%	82.6%	92.5%	80.8%

Among misclassified pixels, agriculture was most often misclassified as forest, and vice versa, for all case studies (with the exception of Peru, where forest was misclassified as both agriculture and wetland; S1 File). Bare soil was often misclassified as agriculture or forest. In Bolivia, which has the highest desert area of our land covers, bare soil and desert were often mistaken for one another. Finally, urban, water, and wetland land covers were all most often misclassified as forest.

### Land cover trends

**Fig 1:**
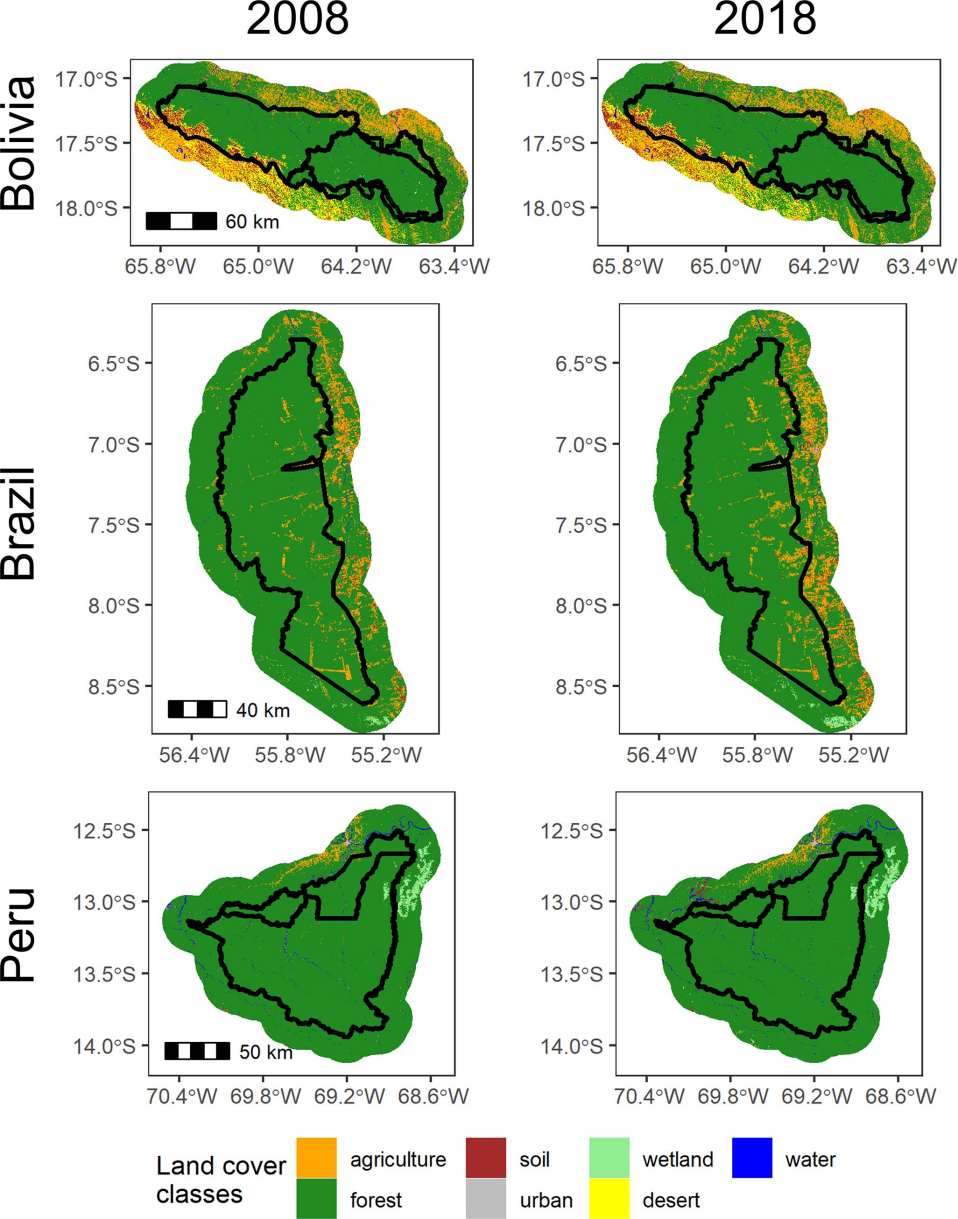
Classified land cover maps for Amboró and Carrasco National Parks in Bolivia, Jamanxim national forest in Brazil, and Tambopata National reserve and bahuaja-Sonene National Park in Peru. Black lines indicate park boundaries, with a 20km external buffer. Rio Novo National Park and the Ambiental Do Tapajós Protected Areas park borders Jamanxim to the west, Rio Grande Valles Crucenos borders Amboró National Park to the southeast, and Madidi National Park borders Bahuaja-Sonene to the east. Maps derived from Landsat 5 (TM), Landsat 7 (ETM+), and Landsat 8 (OLI) imagery, courtesy of the U.S. Geological Survey.

Our maps indicated that all PAs saw some level of deforestation between 2008–2018 and deforestation rates were consistently higher in the buffer regions than in the PAs (Fig 1). Our Brazil site had the highest deforestation rates (% area lost between 2008–2018), at 3.6% within Jamanxim National Forest and 6.7% in the 20km surrounding Jamanxim (Table 3). Deforestation rates were more modest in Amboró (<1%) and Carrasco (<2%) National Parks in Bolivia, though relatively high in the IMNA integrated use region around Amboró (5.0%). Finally, deforestation rates were relatively low within Tambopata National Reserve and Bahuaja-Sonene National Park (<1% for both sites), and slightly higher in the 20km buffer surrounding these Peruvian PAs (2.7%).

**Table 3:** Detected deforestation (2008–2018) between our and Global Forest Change’s land cover maps.

	Our maps		Global Forest Change	
Bolivia	Percent loss per area	Total km^2^ forest lost	Percent loss per area	Total km^2^ forest lost
Carrasco National Park	1.4%	83.5	1.8%	132.4
Amboró National Park	0.4%	18.6	0.6%	28.2
Amboró IMNA	5.0%	84.7	7.8%	131.3
In 20km buffer (including desert deforestation)	7.9%	1169.3		
In 20km buffer (excluding desert deforestation)	6.5%	956.4	6.7%	1003.3
Brazil				
Jamanxim National Forest	3.6%	471.1	5.6%	739.7
In 20km buffer	6.7%	962.9	9.1%	1319.8
Peru				
Tambopata National Reserve	0.8%	24.0	0.8%	22.6
Bahuaja-Sonene National Park	0.4%	39.6	0.4%	42.9
In 20km buffer	2.7%	353.6	5.4%	729.9

**Fig 2:**
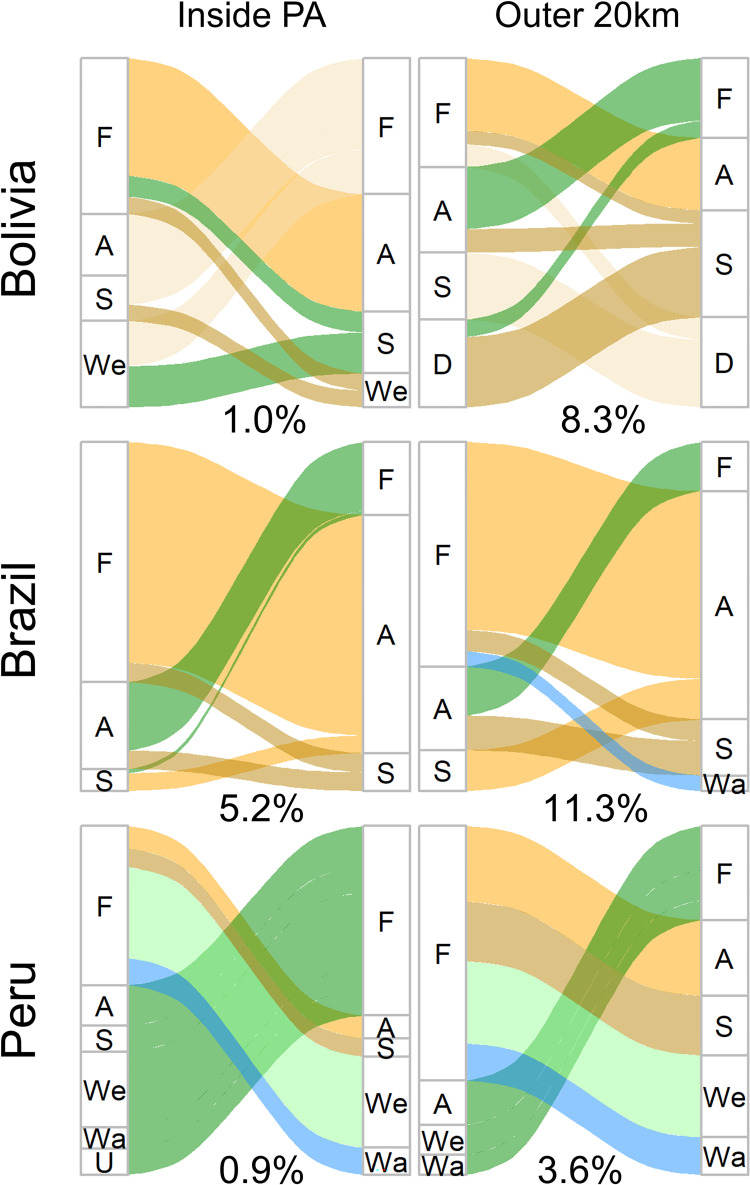
Sankey graphs demonstrating 2008–2018 land cover transitions. Flow widths represent proportions of land area transitioning and colors follow the final land classification assignment. Numbers below each graph represent the percent of pixels within that land area that transitioned. If a transition frequency (i.e. water to urban) accounted for less than 1% of all transitions, we did not graph it, for visual simplicity. Land classes: F: forest, A: agriculture (and pasture), S: bare soil, We: wetland, Wa: water, D: desert, U: urban.

Beyond deforestation, looking at all land cover transitions in our land cover maps gives us insight into land use change patterns. Within Amboró and Carrasco National Parks in Bolivia, 1.0% of pixels transitioned land covers between 2008 and 2018. Of these pixel transitions, 33.1% were forest transitioning to agriculture or bare soil, while 21.7% were agriculture or bare soil transitioning to forest (while 0.56% of the PAs were agriculture or soil in 2008, 0.75% were agriculture or soil in 2018; Fig 2). In the 20km buffer in Bolivia, 8.3% of pixels transitioned between 2008 and 2018, and 32% of these transitions were between bare soil and desert. Otherwise, deforestation and reforestation rates were similar in the buffer area, and proportions of land covers stayed nearly constant between 2008 and 2018 (agriculture and bare soil area only increased by 0.03% of the PAs’ area between 2008 and 2018).

In Jamanxim National Forest in Brazil, 5.2% of pixels transitioned in the PA and 11.3% of pixels transitioned in the 20km buffer between 2008–2018. Conversion of forest to agriculture or bare soil was the most common transition type both within the PA (68.3% of transitions) and in the buffer area (57.0% of transitions). While agriculture and bare soil covered 4% of the PA and 11.6% of the buffer region in 2008, they covered 6.5% of the PA and 16.4% of the buffer region in 2018. A smaller percent of pixels transitioned from agriculture to forest (19.5% in the PA and 13.4% in the buffer). Rates of land use change between other classes were very small. Most of the deforestation occurred on the eastern side of the PA (and it should be noted that there is another PA bordering Jamanxim on the west side).

Inside the boundaries of Tambopata National Reserve and Bahuaja-Sonene National Park in Peru, only 0.9% of pixels transitioned between 2008 and 2018. While 40.0% of land use change here represented deforestation (transitions from forest to other land covers), 45.0% of land use change represented reforestation (transitions to forest). Conversely, 3.6% of pixels in the 20km buffer transitioned between 2008 and 2018, with 63.3% of pixels transitioning from forest to other land classes and 26.3% of pixels transitioning from non-forest land covers to forest. In particular, soil and water areas (combined) increased from 2.2% to 2.9% of the total buffer area between 2008 and 2018. Conversion of forest to agriculture was concentrated around existing agriculture, in the north of Tambopata National Reserve, while conversion from forest to soil and water occurred in the northwest corner of the buffer (there is another PA bordering our case study on the southeast side).

### Comparison between our and GFC’s maps

Our and GFC’s land cover maps show similar general deforestation trends, though GFC tended to record higher levels of deforestation (Table 3). For instance, GFC estimates of deforestation in Jamanxim were 55% higher than our analysis and estimates in the 20km buffer region of the Peru site were twice as high as our estimate. The only region where our maps detected more deforestation than GFC was in the 20km buffer for Bolivia, where we found desert vegetation converted to agriculture (GFC does not consider desert conversions as deforestation). If we do not include desert to agriculture transitions in our calculation of deforestation, GFC has a slightly higher deforestation rate. Our land cover maps show that a very small number of pixels were reforested, with 0.12% of pixels in Brazil, 0.69% in Bolivia, and 0.23% in Peru.

**Fig 3:**
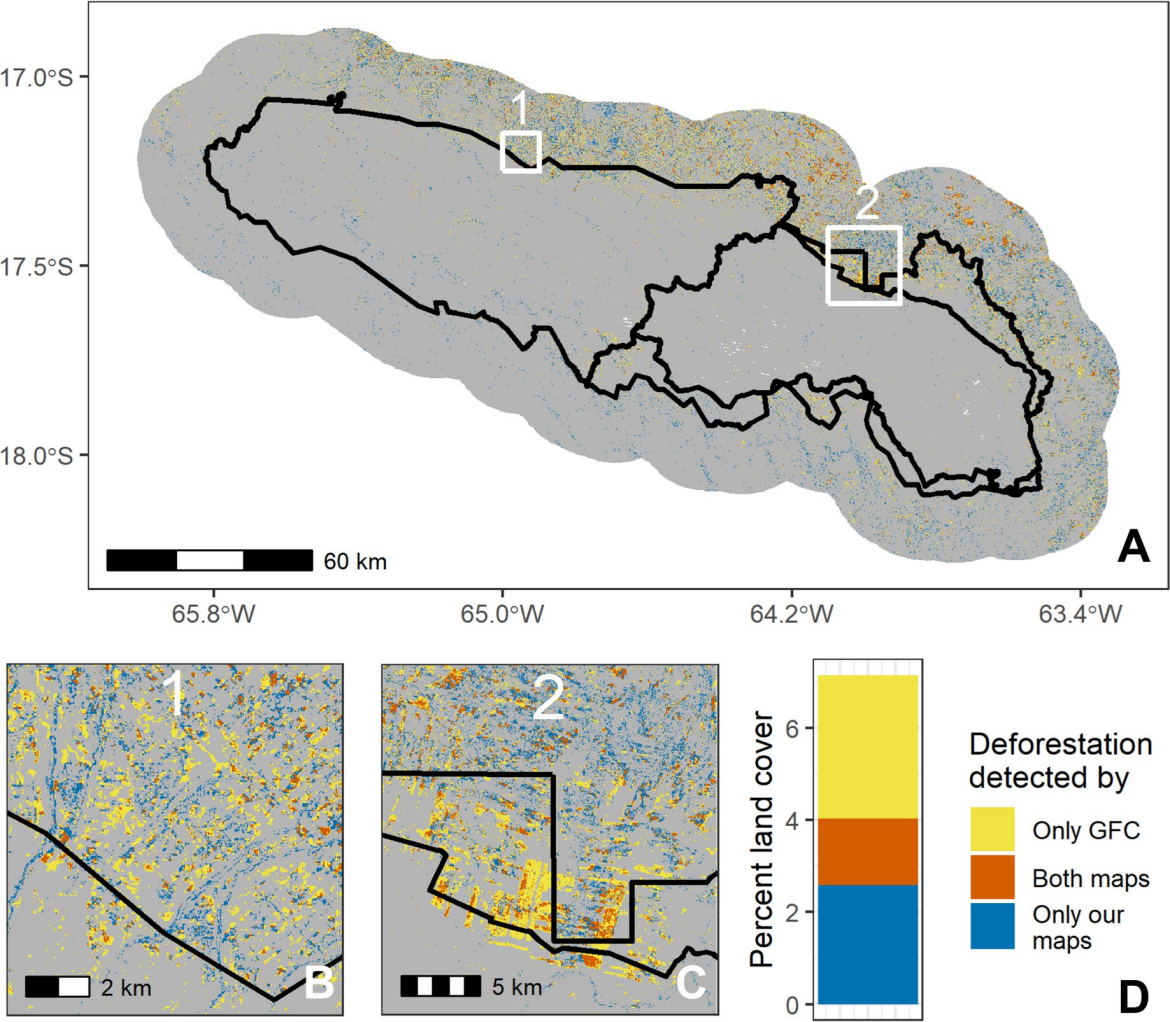
Spatial comparison of our and Global Forest Change’s maps in the Bolivia case study. Panel A shows the entire study area (with the protected area borders in black), panels B and C show two close-up regions (labeled 1 and 2 in panel A), and panel D quantifies types of disagreement by percent area. Grey indicates where both maps agreed there was no land cover change between 2008 and 2018. Our maps were derived from Landsat 5 (TM), Landsat 7 (ETM+), and Landsat 8 (OLI) imagery, courtesy of the U.S. Geological Survey. Global Forest Change source: Hansen/UMD/Google/USGS/NASA.

**Fig 4:**
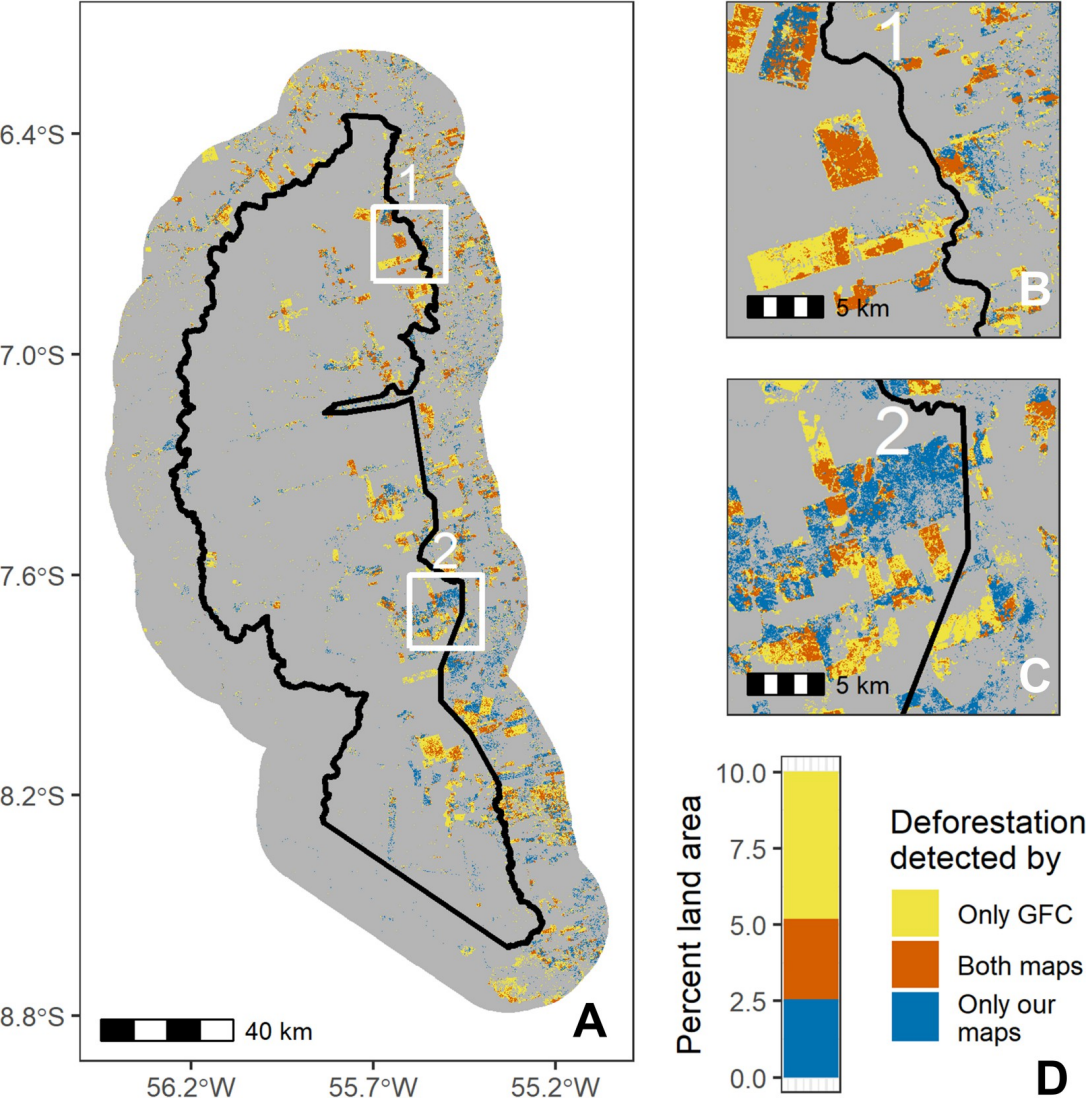
Spatial comparison of our and Global Forest Change’s maps in the Brazil case study. Panel A shows the entire study area (with the protected area border in black), panels B and C show two close-up regions (labeled 1 and 2 in panel A), and panel D quantifies types of disagreement by percent area. Grey indicates where both maps agreed there was no land cover change between 2008 and 2018. Our maps were derived from Landsat 5 (TM), Landsat 7 (ETM+), and Landsat 8 (OLI) imagery, courtesy of the U.S. Geological Survey. Global Forest Change source: Hansen/UMD/Google/USGS/NASA.

**Fig 5:**
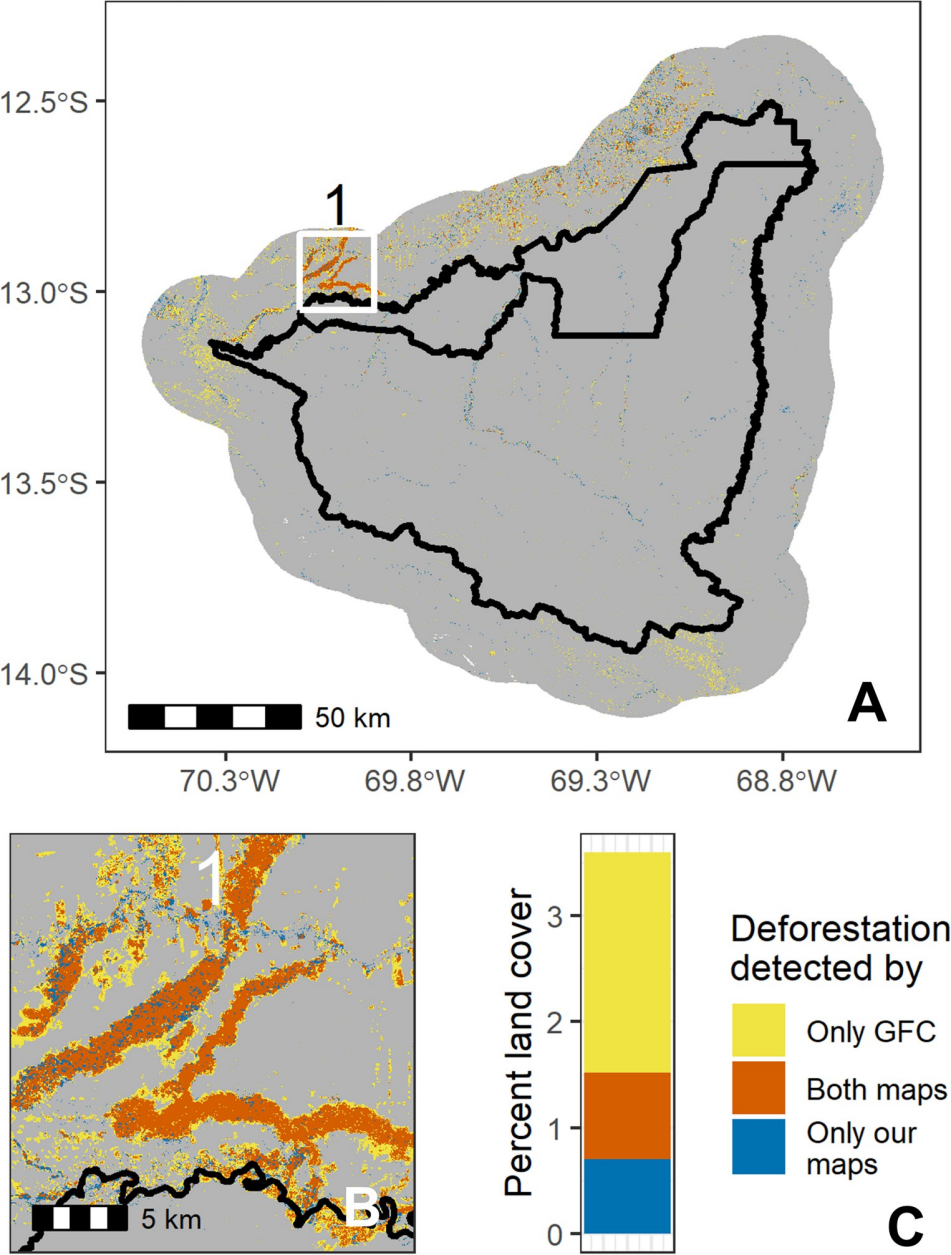
Spatial comparison of our and Global Forest Change’s maps in the Peru case study. Panel A shows the entire study area (with the protected area borders in black), panel B shows a close-up region to demonstrate details, and panel C quantifies types of disagreement by percent area. Grey indicates where both maps agreed there was no land cover change between 2008 and 2018. Our maps were derived from Landsat 5 (TM), Landsat 7 (ETM+), and Landsat 8 (OLI) imagery, courtesy of the U.S. Geological Survey. Global Forest Change source: Hansen/UMD/Google/USGS/NASA.

Spatial overlap analysis further showed disagreement between our and GFC’s land cover maps (Figs 3–5). In the Brazil site, 7.4% of pixels disagreed about whether there was deforestation between 2008–2018 (quantity difference: 2.3%; allocation difference: 5.1%), compared to 5.7% for Bolivia (quantity difference: 0.5%; allocation difference: 5.2%; excluding desert points) and 2.8% for Peru (1.4% quantity difference; 1.4% allocation difference). Of the pixels in which the two land cover maps disagreed, most pixels were areas where GFC detected deforestation where our maps did not (Figs 3–5). However, our land cover maps also revealed 229 km^2^ of desert deforestation in Bolivia (not included in Fig 3), which was not detected by GFC maps (this comprises 12% of land cover disagreements when desert deforestation is included in the analysis).

### Dissimilarity analysis

**Fig 6:**
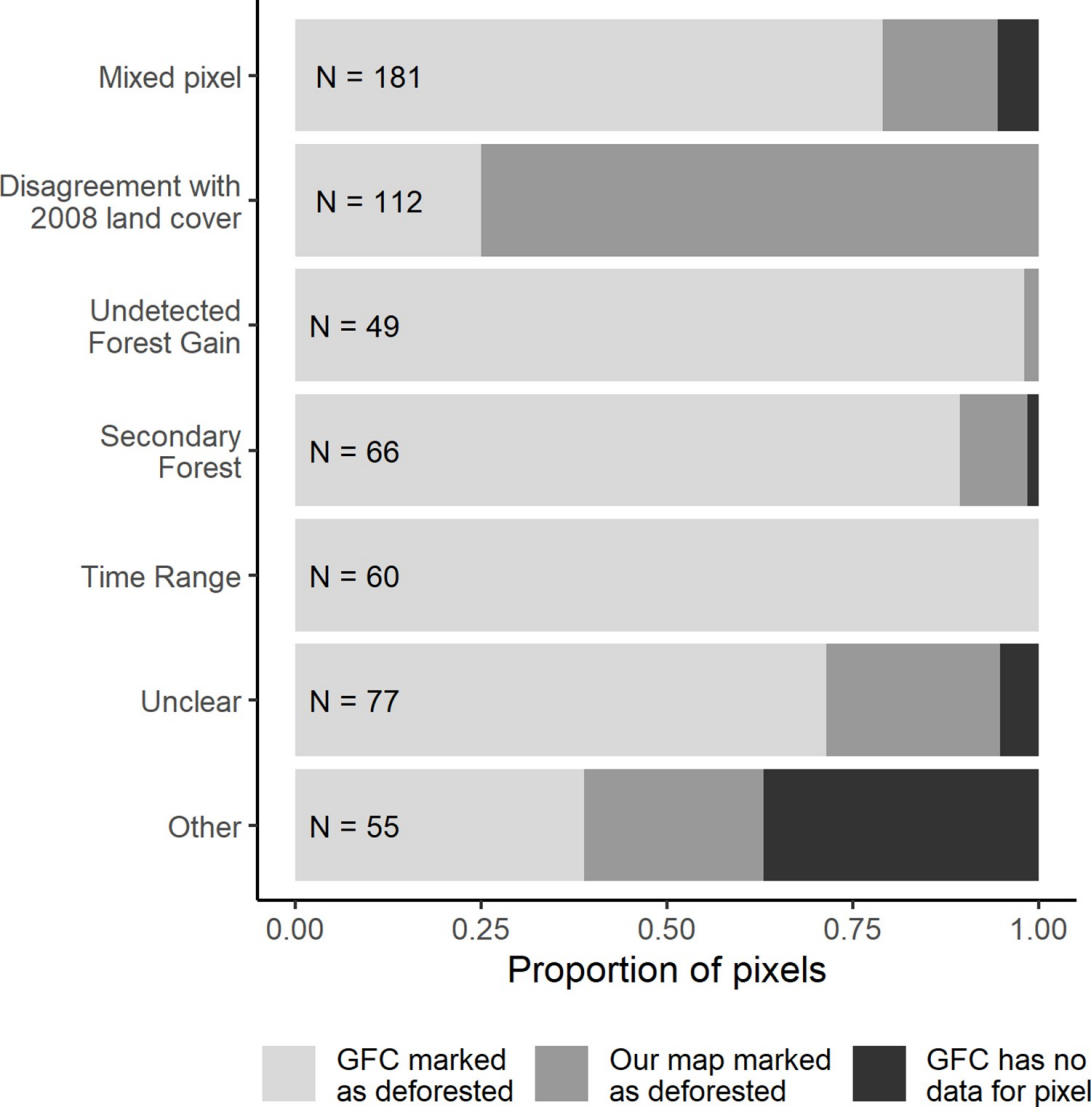
Explanations for dissimilarity among our and Global Forest Change’s maps. N represents the sampling points (out of 600 total) that fell into each explanation of dissimilarity (excluding desert points). We additionally show the proportion of pixels Global Forest Change marked as deforested where our maps detected no change, pixels that our maps marked as deforested where GFC detected no change, and pixels where GFC had no data for forest cover in 2000 or forest loss.

For the pixels in which our land cover map disagreed with GFC’s, we found 6 main explanations for dissimilarity: Mixed Pixels, Disagreement in 2008 Land Cover, Undetected Forest Gain, Secondary Forest, Time Range Issues, and Unclear (Fig 6). We also identified some less common explanations, which we list below. More detail on the dissimilarity analysis results for each study site can be found in S1 Table.

**Mixed Pixels** (including sparse forest) refer to pixels in which two land covers occurred (with neither land cover covering more than 80% of the pixel area). Mixed pixels were the biggest source of dissimilarity between the two map products, explaining 30.2% of the dissimilar points. Of mixed pixel disagreements, 79% were half forest and half non-forest cover that GFC classified as “deforested” and our maps classified as forest (Fig 6).

The second most common explanation for dissimilarity was a **Disagreement in 2008 Land Cover,** comprising 19% of the total dissimilar pixels. Differences in classification of 2008 land cover caused disagreements in the transition type, even if the maps agreed on the 2018 land cover. For example, if one map determined a pixel to be forested in 2008 while the other determined it to be non-forest, even if they both determined the land cover in 2018 as non-forest, one would mark the pixel as “deforested” while the other would mark it as “no change.” Because we did not have consistent high-resolution imagery for 2008, we couldn’t further inspect dissimilarity in 2008. However, we did find that our maps classified 75% of pixels in this category as “deforested” while GFC classified them as “no change” (with GFC specifically indicating that pixels were deforested in 2008 and remained deforested in 2018; Fig 6).

Some dissimilar points were caused by **Undetected Forest Gain**, specifically that some pixels showed evidence of once being deforested, but in 2018 appeared regrown (with reflectance patterns similar to mature forests and with little if any visible fluctuation of phenology of these sites between seasons). This problem was specific to the GFC maps, in which forest gain data is only available for 2000–2012, and possibly not very accurate for that time period. Therefore, these pixels were generally classified as “deforested” by GFC’s maps but as “no change” by our maps. Undetected forest gain was most common in Brazil, accounting for 11% of all dissimilar pixels there. Peru also had 9% dissimilarity due to undetected forest gain, while Bolivia’s rates were 4.5%.

**Secondary Forest** refers to pixels which had been deforested but were in the process of reforesting (evidenced by looking forward and back in time with PlanetLabs). Secondary forest pixels caused confusion for the classification algorithms, which either classified them as deforested or forested. This category differs from the undetected forest gain category because the vegetation in secondary forest pixels was not as fully structured (less mature) as forested sites. Within this category of dissimilar points, GFC classified secondary forest as deforested where we classified it as forest 89% of the time (Fig 6). Classifying secondary forest as deforestation may also reflect the limitation of GFC to not register forest gain after 2012. Out of all dissimilar pixels, 11% were caused by confusion over secondary forest.

We also found disagreements between land cover classification due to **Time Range Issues**, or a mismatch between the capturing period of our and GFC’s maps. We captured satellite data between mid-May and October in 2008 and 2018, prioritizing earlier months in each year. GFC composites also pulled satellite data from growing season months, but GFC does not specify the specific time range or how pixels are chosen from that range. We found that deforestation that occurred after May or June was generally not captured in our maps but was captured by GFC, thus causing a discrepancy. 10% of all dissimilar pixels were caused by time range issues.

For pixels classified as **Unclear,** it was apparent from high resolution images that an area was forested or deforested, but one of the remote sensing products misclassified it without a clear explanation why. This may ultimately be due to classification errors, and comprised 13% of the dissimilar pixels (with the least amount of unclear pixels in the Brazil site). In general, GFC more often misclassified pixels that were forest (with no evidence of once being deforested) as deforested, while our maps more often misclassified deforested pixels as forested. Errors were more often attributed to GFC in Bolivia, evenly distributed in Brazil, and more often attributed to our maps in Peru. In total, GFC had only slightly more errors than our maps (comprising 54% of errors).

Other less common reasons for misclassification included mistaking a natural feature for agriculture or deforestation (Fig 6). Mountain tops, wetlands, and rivers were all misclassified at modest rates. Because wetland was one of our classes, our classification did not commonly mistake wetlands for agriculture, while GFC did so more often. Rivers changing course and leaving sandy deposits triggered “deforestation” in both datasets. In 36% of instances in this “other” category, GFC lacked data on forest cover and loss, and this was most common in Bolivia. Additionally, while not common, it was possible for pixels to fall into multiple categories of dissimilarity explanations (for example mixed pixel and time range).

## Discussion

This study elucidates deforestation trends within and around three PA complexes in the Amazon Basin using author-generated land cover maps and the GFC dataset. Our created land cover maps have high overall accuracy and generally high accuracy for specific land cover classes (Table 2). While water and urban areas show poor accuracy values for some case studies, these land covers represent very small proportions of the study area and should not pose a concern (though we do not recommend use our maps to specifically track changes to water or urban areas). Using our created maps, we demonstrate varying land use change patterns in our case studies, such as high conversion of forest to agriculture in and around Jamanxim National Forest in Brazil, and high conversion of forest to water and bare soil classes near the northern border of Tambopata National Reserve in Peru. By comparing trends between our maps and the GFC dataset, we find that GFC overestimates deforestation while our maps underestimate it. Our study provides novelty by demonstrating that these biases occur due to different treatment of mixed pixels, poor recognition of forest regrowth by GFC, and a propensity for GFC to misclassify forest as deforested land and our maps to misclassify deforested land as forest. We suggest, therefore, that true deforestation rates likely fall between the estimates from our and GFC’s land cover maps (Table 3).

### Land change trends in the context of the Amazon Basin

All three case study sites saw deforestation between 2008 and 2018, though rates and likely drivers differed. In Bolivia, while in the late twentieth century deforestation rates in Amboró and Carrasco National Parks were growing rapidly, we have found that rates have since stayed steady or decreased. For context, the rate of deforestation in Carrasco National Park grew from 2 km^2^/yr to 35.2 km^2^/yr between the early 1980s and 2000s [35], but we found a rate of 10.8 km^2^/yr (taking an average of rates from our and GFC’s maps). Similarly, deforestation rates in Amboró National Park have risen only slightly, from 1.8 km^2^/yr in the early 2000s to 2.3 km^2^/yr in our study, and have decreased in the Amboró IMNA site, from 14.3km^2^/yr to 10.8km^2^/yr. The conversion of primary forest to agriculture and bare soil that we detect in these Bolivian parks (Fig 2) likely reflects the expansion of mechanized agriculture, cattle ranching, and small-scale agriculture [62]. It is also notable that deforestation rates are higher in the integrated use (IMNA) site around Bolivia, and in fact we found higher deforestation in the IMNA site than in the 20km buffer around the PAs. While the Amboró IMNA area has fewer restrictions on land use to allow local communities to use the forest to support their livelihoods (Bucklin 2010), this is an area of conservation interest and deforestation here should be monitored in future years.

The high rates of deforestation we observed in Jamanxim National Forest align with understanding of forest loss in this area. Previous studies have found high forest loss and fragmentation in Jamanxim relative to other PAs in the Brazilian Amazon [11, 63] https://www.zotero.org/google-docs/?8UM1DQ, and that Pará, the state where Jamanxim is located, had some of the highest rates of deforestation in Brazil throughout the 2000s [19]. Furthermore, this region has experienced increased deforestation since the Forest Code was amended in 2012, which reduced environmental protections [64], and deforestation has continued to rise since the end of our study period [65]. In Jamanxim, a rise in medium- and large-scale deforestation processes, such as diverse agricultural activities and intense cattle ranching, may contribute to the forest loss we detected [63].

However, Jamanxim also had the highest rates of reforestation among our study sites. Over our study period, 377 km^2^ of forest regrew and, for context, 148,765 km^2^ of secondary forests recovered between 1986–2018 across the Brazilian Amazon [66]. Some of this forest regrowth may reflect areas in cycles of regrowth and harvesting (repeated logging in the same area), which we observed while doing the dissimilarity analysis. Forest regrowth may also occur on agricultural land, for instance where degraded pastures are abandoned and forests subsequently regrow [63].

Deforestation rates in the Peru site were modest compared to the other two study sites. The increase in bare soil and water, particularly in the buffer area north of Tambopata National Reserve (Fig 2), may reflect the creation of small-scale gold mines. The conversion of forests to artisanal gold mines is a regional trend: across Madre de Dios, conversion of forests to gold mines occurred at a rate of 44.4 km^2^/year from 1999–2016 [67]. Similarly, we found around 54.2 km^2^/year of forest loss between 2008–2018 in the 20km buffer region around Tambopata (again taking the average of deforestation rates from our and GFC’s maps).

### Differences between datasets

While over 94% of the study sites’ areas were similarly classified by our land cover maps and GFC, the differences are non-negligible and represent over 4000 km^2^ of land. Around 2000 km^2^ of this area of disagreement is found in and around Jamanxim National Forest in Brazil, representing a significant proportion (15%) of the Brazil study site.

Much of the discrepancy between land cover maps reflects instances where GFC mapped deforestation where we did not (Figs 3–5), leading to consistently higher deforestation rates in the GFC maps (Table 3). Other studies have also found that GFC overestimates deforestation, including in dry tropical forests of Costa Rica [22] and rainforests of Gabon [61]. Cunningham et al. (2019) noted that this effect was particularly prevalent at high elevations, which they postulated was caused by high cloud cover and terrain shadowing. Galiatsatos et al. (2020) also found that GFC overestimated forest loss in Guyana between 2015 and 2017, though they additionally found that GFC overestimated tree canopy cover percent in 2000—thus, total forest cover in 2017 was roughly accurate as these two biases averaged each other out. However, other studies have found the opposite effect. For example, smaller-scale disturbances can be under-detected by GFC in the Amazon [68], and precipitation regimes (dry versus humid tropics) may affect the direction of bias in the GFC product [22].

There are many explanations why land cover maps differ from each other, including known limitations, algorithm parameters, or differences with input data or training data [29]. For instance, image acquisition dates of each land cover map are inherent differences between map methodologies which caused moderate disagreement between our and GFC’s land cover maps. We found that GFC registered deforestation occurring in later months of the image acquisition year than our maps, thus logging slightly more deforestation at the tail end of the year. Other studies have encountered similar temporal constraints [68], and users should be aware of this when comparing land cover maps. Additionally, some studies have found errors in the year GFC marks an area as deforested [20, 23], though we did not find this error in our study.

Another inherent limitation with GFC is a lack of data on reforestation, especially after 2012, which can have large impacts on results interpretation. For instance, while we found moderate rates of deforestation inside the Bolivian and Peruvian PAs, we found similar rates of reforestation (Fig 2), thus resulting in little changes in total forest cover. Rates of reforestation in the Brazil PA, while much lower than deforestation rates, were still sizeable, representing more reforested land than in the Bolivia and Peru study sites. This highlights that not all land classified as deforested by GFC has remained in that state (Table 3). We also found a weakness in GFC’s ability to detect reforestation before 2012. In the dissimilarity analysis, there were many pixels that disagreed on the land cover in 2008, specifically where our classification detected forest while GFC detected deforestation with no forest gain. We suspect that many of the disagreements may be caused by poor detection of reforestation by GFC. Sannier et al. (2016) [61] also found that detection of reforestation between 2000–2012 was much less accurate than detection of deforestation, perhaps due to the difference in spectral signals that these events create (one gradual and the other generally very stark).

We also found that GFC tended to classify areas as deforested more readily than our maps, which is reflected within mixed and misclassified (unclear) pixels in our dissimilarity analysis. This likely indicates differing sensitivity of our and GFC’s classification algorithms in detecting forest or non-forest land covers, which may occur for a variety of reasons. Phenological differences due to image acquisition dates, for instance, can affect classification performance [69, 70] https://www.zotero.org/google-docs/?DwUAwS. Disagreement may also be attributed to the training data collection process, which has high potential for human error [71]. Finally, input imagery into our classification may also cause differences, especially if certain imagery more distinctively distinguishes between land covers. While both land cover maps utilized Landsat imagery and decision tree (random forest) classifiers, we included other input variables to our classification, namely EVI and seasonal change in EVI, which may have changed prediction potential. Without a more systematic study on these factors internal to the classification scheme, it’s difficult to say to what extent each of these factors has caused the differences we have seen. In summary, considering that GFC overestimates deforestation while we underestimate it, we suggest that true deforestation rates lie somewhere between our two estimates (Table 3), and again note that these rates do not account for reforestation.

### Broader implications and conclusions

When land use change researchers are faced with the choice of which spatial dataset to utilize, we encourage them to keep in mind how different land cover maps can bias understandings. We found that our land cover maps and the GFC maps have specific strengths and weaknesses. Our maps detect forest regrowth and are more sensitive to secondary forest, which could clue researchers and land managers to areas that may be reforesting or in a cycle of logging and regrowth. We also find a moderate amount of conversion of desert to agriculture, which has important implications for conservation, but is not detected by GFC because desert ecosystems are generally not considered “forest” by this dataset. Details about specific land cover transitions that our maps provide also ties to more local dynamics, for example better guiding local decision-making and enforcement, and helping identify local deforestation drivers. On the other hand, GFC is better suited for more large-scale analyses because of its global scope. Furthermore, a key benefit of GFC over our land cover maps is its ability to detect land change in discrete years rather than solely between 2008 and 2018, making it more useful when information on annual trends is needed. Thus, researchers should consider which land cover map best suits their research questions.

Broadly, our results demonstrate the challenges in creating land cover maps. Land use is extremely complex, and different sets of conditions, history, and dynamics are difficult to interpret from pixelated data [72]. Each set of land cover maps contains its own limitations and biases, which should not overshadow the value of these products, but rather guide more intentional usage. In the case of GFC, especially given its wide use, we hope our results will guide future use and communication and retroactively aid in the interpretation of previous studies, thus better supporting policy and management applications.

In conclusion, this study gives a transparent understanding of deforestation trends in three tropical forest PAs and raises awareness on land cover map biases and limitations by demonstrating the impact that land cover map choice can have on understanding land use change. Detailed understanding of these transitions is key to monitoring deforestation and studying deforestation dynamics, thereby helping establishing conservation plans that can best conserve these areas of incredible ecological, cultural, and biophysical importance [31, 32].

## Supporting information

S1 File Sample confusion matrixes and estimated confusion matrixes for all case studies. (DOCX)

S1 Table Categorized results from the dissimilarity analysis. (DOCX)
