# Supplementary material for: Biases and limitations of Global Forest Change and author-generated land cover maps in detecting deforestation in the Amazon
Source: PLoS One. 2022 Jul 6;17(7):e0268970. doi: 10.1371/journal.pone.0268970 (PMC9258877; doi:10.1371/journal.pone.0268970)
Supplement: S1 File — (DOCX) [file pone.0268970.s001.docx]

# **S1 File: Sample confusion matrixes and estimated confusion matrixes for all case studies**

**Sample confusion matrix for the Bolivia case study**. The cell values represent pixel counts, with each pixel equating 900m^2^. The population total is the number of pixels in each land cover for the entire study area, with which we calculated the estimated population matrix (S2 Table). Reference land covers are on the x axis and predicted land covers are on the y axis for this confusion matrix and confusion matrices in S2-6.

|  | 1 | 2 | 3 | 4 | 5 | 6 | 7 | Row Total | Population Total |
| --- | --- | --- | --- | --- | --- | --- | --- | --- | --- |
| 1: Agriculture | 10406 | 661 | 121 | 19 | 4 | 129 | 10 | 11350 | 4720735 |
| 2: Forest | 792 | 18929 | 86 | 28 | 3 | 226 | 39 | 20103 | 22681043 |
| 3: Bare soil | 250 | 175 | 4745 | 15 | 0 | 262 | 34 | 5481 | 1747545 |
| 4: Urban | 99 | 71 | 45 | 940 | 0 | 3 | 3 | 1161 | 112912 |
| 5: Water | 20 | 10 | 0 | 0 | 47 | 0 | 2 | 79 | 2109 |
| 6: Desert | 66 | 119 | 205 | 0 | 0 | 4485 | 1 | 4876 | 2401069 |
| 7: Wetland | 45 | 121 | 60 | 4 | 0 | 8 | 2146 | 2384 | 190396 |
| Column Total | 11678 | 20086 | 5262 | 1006 | 54 | 5113 | 2235 |  |  |

**Estimated population confusion matrix for the Bolivia case study.** The cell values represent percent land area.

|  | 1 | 2 | 3 | 4 | 5 | 6 | 7 | Row Total |
| --- | --- | --- | --- | --- | --- | --- | --- | --- |
| 1: Agriculture | 13.587 | 0.860 | 0.158 | 0.025 | 0.005 | 0.168 | 0.013 | 14.816 |
| 2: Forest | 2.805 | 67.041 | 0.305 | 0.099 | 0.011 | 0.800 | 0.138 | 71.199 |
| 3: Bare Soil | 0.250 | 0.175 | 4.749 | 0.015 | 0.000 | 0.262 | 0.034 | 5.486 |
| 4: Urban | 0.030 | 0.022 | 0.014 | 0.287 | 0.000 | 0.001 | 0.001 | 0.354 |
| 5: Water | 0.002 | 0.001 | 0.000 | 0.000 | 0.004 | 0.000 | 0.000 | 0.007 |
| 6: Desert | 0.102 | 0.1839 | 0.317 | 0.000 | 0.000 | 6.933 | 0.002 | 7.537 |
| 7: Wetland | 0.011 | 0.0303 | 0.015 | 0.001 | 0.000 | 0.002 | 0.538 | 0.598 |
| Column Total | 16.787 | 68.313 | 5.557 | 0.427 | 0.020 | 8.167 | 0.726 |  |

**Sample confusion matrix for the Brazil case study.** The cell values represent pixel counts, and each pixel is 900m^2^.

|  | 1 | 2 | 3 | 4 | 5 | 7 | Row Total | Population Total |
| --- | --- | --- | --- | --- | --- | --- | --- | --- |
| 1: Agriculture | 7729 | 334 | 141 | 24 | 4 | 3 | 8235 | 3153484 |
| 2: Forest | 575 | 24588 | 48 | 7 | 4 | 19 | 25241 | 26876745 |
| 3: Bare soil | 524 | 129 | 2254 | 4 | 0 | 0 | 2911 | 434305 |
| 4: Urban | 29 | 63 | 17 | 560 | 0 | 0 | 669 | 26427 |
| 5: Water | 1 | 6 | 0 | 0 | 174 | 0 | 181 | 153424 |
| 7: Desert | 3 | 35 | 0 | 0 | 0 | 857 | 895 | 106295 |
| Column Total | 8861 | 25155 | 2460 | 595 | 182 | 879 |  |  |
|  |  |  |  |  |  |  |  |  |

**Estimated population confusion matrix for the Brazil case study.** Cell values represent percent land area.

|  | 1 | 2 | 3 | 4 | 5 | 7 | Row Total |
| --- | --- | --- | --- | --- | --- | --- | --- |
| 1: Agriculture | 9.625 | 0.416 | 0.176 | 0.030 | 0.005 | 0.004 | 10.255 |
| 2: Forest | 1.991 | 85.141 | 0.166 | 0.024 | 0.014 | 0.066 | 87.402 |
| 3: Bare soil | 0.254 | 0.063 | 1.094 | 0.002 | 0.000 | 0.000 | 1.412 |
| 4: Urban | 0.004 | 0.008 | 0.002 | 0.072 | 0.000 | 0.000 | 0.086 |
| 5: Water | 0.003 | 0.017 | 0.000 | 0.000 | 0.480 | 0.000 | 0.499 |
| 7: Wetland | 0.001 | 0.014 | 0.000 | 0.000 | 0.000 | 0.331 | 0.346 |
| Column Total | 11.878 | 85.658 | 1.438 | 0.128 | 0.499 | 0.401 |  |

**Sample confusion matrix for the Peru case study.** The cell values represent pixel counts, with pixels equating 900m^2^.

|  | 1 | 2 | 3 | 4 | 5 | 6 | 7 | Row Total | Population Total |
| --- | --- | --- | --- | --- | --- | --- | --- | --- | --- |
| 1: Agriculture | 4320 | 160 | 14 | 14 | 34 | 0 | 4 | 4546 | 475662 |
| 2: Forest | 136 | 26038 | 37 | 14 | 54 | 0 | 67 | 26346 | 29107632 |
| 3: Bare soil | 80 | 123 | 1037 | 4 | 49 | 1 | 47 | 1341 | 157567 |
| 4: Urban | 36 | 19 | 6 | 691 | 0 | 0 | 0 | 752 | 50086 |
| 5: Water | 12 | 24 | 12 | 0 | 3148 | 0 | 0 | 3196 | 518342 |
| 6: Desert | 0 | 3 | 0 | 0 | 0 | 54 | 0 | 57 | 4110 |
| 7: Wetland | 8 | 96 | 37 | 3 | 0 | 0 | 1783 | 1927 | 363083 |
| Column total | 4592 | 26463 | 1143 | 726 | 3285 | 55 | 1901 |  |  |

**Estimated population confusion matrix for the Peru case study.** Cell values represent percent land area.

|  | 1 | 2 | 3 | 4 | 5 | 6 | 7 | Row Total |
| --- | --- | --- | --- | --- | --- | --- | --- | --- |
| 1: Agriculture | 1.474 | 0.055 | 0.005 | 0.005 | 0.012 | 0.000 | 0.001 | 1.551 |
| 2: Forest | 0.490 | 93.78 | 0.133 | 0.050 | 0.195 | 0.000 | 0.241 | 94.886 |
| 3: Bare soil | 0.031 | 0.047 | 0.397 | 0.002 | 0.019 | 0.000 | 0.018 | 0.514 |
| 4: Urban | 0.008 | 0.004 | 0.001 | 0.150 | 0.000 | 0.000 | 0.000 | 0.163 |
| 5: Water | 0.006 | 0.013 | 0.006 | 0.000 | 1.664 | 0.000 | 0.000 | 1.690 |
| 6: Desert | 0.000 | 0.001 | 0.000 | 0.000 | 0.000 | 0.013 | 0.000 | 0.013 |
| 7: Wetland | 0.005 | 0.059 | 0.023 | 0.002 | 0.000 | 0.000 | 1.095 | 1.184 |
| Column Total | 2.013 | 93.955 | 0.566 | 0.209 | 1.889 | 0.013 | 1.356 |  |
