# Supplementary material for: Biases and limitations of Global Forest Change and author-generated land cover maps in detecting deforestation in the Amazon
Source: PLoS One. 2022 Jul 6;17(7):e0268970. doi: 10.1371/journal.pone.0268970 (PMC9258877; doi:10.1371/journal.pone.0268970)
Supplement: S1 Table — (DOCX) [file pone.0268970.s002.docx]

**S1 Table:** **Categorized results from the dissimilarity analysis.** We include the number of pixels for each case study site that fell into each reason for dissimilarity and how it was classified by the two land cover maps (detected as deforested by Global Forest Change (GFC) and no change in our maps and vice versa). We also include the number of pixels for which GFC had no data.

|  | **Bolivia** | | | **Brazil** | | | **Peru** | | |
| --- | --- | --- | --- | --- | --- | --- | --- | --- | --- |
| Reason for dissimilarity | Deforested in GFC maps | Deforested in our maps | GFC has no data | Deforested in GFC maps | Deforested in our maps | GFC has no data | Deforested in GFC maps | Deforested in our maps | GFC has no data |
| Mixed pixels (including sparse forest) | 43 | 23 | 8 | 48 | 1 | 1 | 52 | 4 | 1 |
| Disagree-  ment in 2008 imagery | 13 | 28 | 0 | 7 | 45 | 0 | 8 | 11 | 0 |
| Time Range Issues | 11 | 0 | 0 | 15 | 0 | 0 | 34 | 0 | 0 |
| Secondary Forest | 9 | 4 | 1 | 36 | 1 | 0 | 14 | 1 | 0 |
| Undetected Forest Gain | 8 | 1 | 0 | 22 | 0 | 0 | 18 | 0 | 0 |
| Unclear | 13 | 14 | 0 | 14 | 1 | 2 | 28 | 4 | 1 |
| Others (Mountain top, wetland, river) | 3 | 8 | 13 | 6 | 1 | 0 | 6 | 10 | 8 |
| Total pixels (N for each PA = 200) | 100 | 78 | 22 | 149 | 49 | 3 | 160 | 30 | 10 |
